# Oligomeric Status and Nucleotide Binding Properties of the Plastid ATP/ADP Transporter 1: Toward a Molecular Understanding of the Transport Mechanism

**DOI:** 10.1371/journal.pone.0032325

**Published:** 2012-03-16

**Authors:** Aurélien Deniaud, Pankaj Panwar, Annie Frelet-Barrand, Florent Bernaudat, Céline Juillan-Binard, Christine Ebel, Norbert Rolland, Eva Pebay-Peyroula

**Affiliations:** 1 CEA, Institut de Biologie Structurale Jean-Pierre Ebel, Grenoble, France; 2 CNRS, Institut de Biologie Structurale, Grenoble, France; 3 Université Joseph Fourier Grenoble 1, Institut de Biologie Structurale, Grenoble, France; 4 CNRS, Laboratoire de Physiologie Cellulaire & Végétale, UMR5168, Grenoble, France; 5 CEA, LPCV, Institut de Recherches en Technologies et Sciences pour le Vivant, Grenoble, France; 6 Université Joseph Fourier Grenoble 1, LPCV; 7 INRA, LPCV, UMR1200, Grenoble, France; University of Cambridge, United Kingdom

## Abstract

**Background:**

Chloroplast ATP/ADP transporters are essential to energy homeostasis in plant cells. However, their molecular mechanism remains poorly understood, primarily due to the difficulty of producing and purifying functional recombinant forms of these transporters.

**Methodology/Principal Findings:**

In this work, we describe an expression and purification protocol providing good yields and efficient solubilization of NTT1 protein from *Arabidopsis thaliana*. By biochemical and biophysical analyses, we identified the best detergent for solubilization and purification of functional proteins, LAPAO. Purified NTT1 was found to accumulate as two independent pools of well folded, stable monomers and dimers. ATP and ADP binding properties were determined, and Pi, a co-substrate of ADP, was confirmed to be essential for nucleotide steady-state transport. Nucleotide binding studies and analysis of NTT1 mutants lead us to suggest the existence of two distinct and probably inter-dependent binding sites. Finally, fusion and deletion experiments demonstrated that the C-terminus of NTT1 is not essential for multimerization, but probably plays a regulatory role, controlling the nucleotide exchange rate.

**Conclusions/Significance:**

Taken together, these data provide a comprehensive molecular characterization of a chloroplast ATP/ADP transporter.

## Introduction

Membrane nucleotide transporters play a crucial role in chloroplasts, allowing energy in the form of ATP to be imported when it cannot be directly produced by photophosphorylation (*i.e.* at night). These transporters have also been proposed to play a key role in supplying ATP-dependent reactions (such as starch and fatty acid biosynthesis) in non-photosynthetic plastids, with cytosolic ATP [1]. However these transporters still remain poorly characterized at the molecular level. The first bottleneck limiting biochemical characterization of membrane transporters remains their production in heterologous systems. To enhance the success rate for production of recombinant membrane proteins, a parallelized approach was set-up using six different expression systems. One of the aims of this project was to produce the Chloroplast ATP/ADP transporter 1 (NTT1), a 60 kDa transporter from *Arabidopsis thaliana*, located in the inner membrane of the chloroplast envelope. NTT1 contains between eleven and twelve predicted trans-membrane helices and exchanges ATP for ADP [2], [3]. This transporter is completely different from the mitochondrial ATP/ADP carrier which has only six transmembrane helices and exchanges ADP to ATP in an electrogenic way, as reviewed in [4]. During import into the chloroplast envelope, the N-terminus of NTT1 (containing the chloroplast targeting sequence) needs to be cleaved to release the functional, folded mature form of the transporter [2], [5]. Producing an active form of NTT1 in heterologous systems thus requires expression of the corresponding mature protein. In the specific case of NTT1 from *Arabidopsis thaliana*, three expression systems allowed the production of functional NTT1: *E. coli* [6], *L. lactis* [7] and *A. thaliana*.

The production of functional plastid NTT1 in *E. coli* was originally reported by Neuhaus and co-workers [2]. Several charged residues of NTT1 were identified to be essential for steady-state ATP/ADP exchange [8]. Similar studies have more recently focused on the characterization of bacterial NTT1 homologues [3], [9]. A general model for transport of both bacterial and plastidial NTT1-type ATP/ADP transporters has emerged, suggesting that these proteins perform a counter-exchange of ADP+Pi for ATP [3]. These recent reports also provided the first descriptions of purification procedures for recombinant NTT1 proteins produced in bacteria [3], [9]. Using recombinant NTT1 produced either as a mature form of the protein or fused with Mistic [6], we recently got first insights into the oligomeric status of this transporter. In this study, the biochemical properties of this transporter were further characterized. NTT1 was expressed in *E. coli*, purified and studied in a detergent solution. A combination of biophysical and biochemical approaches were used to study the protein at the molecular level.

## Materials and Methods

### Materials

Detergents were obtained from Anatrace, all other chemicals from Sigma.

### Molecular cloning and DNA manipulation

The main construct used in this study is described in detail in [6] and corresponds to Uniprot sequence number Q39002 lacking the first 79 residues corresponding to the N-terminal transit peptide, but containing an N-terminal His-tag and a C-terminal *StreptagII*. Mutants K155E, K155R, E245K, K527E, K527R and the C-terminal deletion were generated with the primers listed in table S1, using the Quick Change Site Directed Mutagenesis kit (Agilent Technologies). The C-terminal Mistic and GFP fusions were introduced in a two-step PCR [10]. The first PCR step was performed with Pfu polymerase (New England Biolabs) to amplify Mistic- or GFP-coding cDNA using primers MisticCterfor, MisticCterrev, GFPfor and GFPrev (Table S1). The purified PCR products were then used as primers in a Quick Change PCR (Agilent), in order to insert Mistic or GFP coding sequences into the pDEST-NTT1 template vector.

### Expression and purification

The different proteins used in this study were expressed and purified using the approach described in [6]. Briefly, the purification procedure consisted in a solubilization step with 1% laurylamidodimethylpropylaminoxyde (LAPAO) followed by an Immobilized-Metal Affinity Chromatography (IMAC) using Ni-NTA resin (Qiagen). After desalting, IMAC-purified proteins were incubated overnight with *Strep*-Tactin beads (IBA). After washing, proteins were eluted in 20 mM Tris pH 8, 100 mM NaCl, 0.1% (w∶v) LAPAO, 3 mM desthiobiotin. The peak fraction was used without any prior buffer exchange for analytical ultracentrifugation analyses. For tryptophan fluorescence measurements, buffer was exchanged just before use for 20 mM Tris pH 8, 10 mM NaCl, 0.1% (w∶v) LAPAO using a PD10 desalting column (GE-Healthcare). Size exclusion chromatography (SEC) experiments were performed on a 20 mL analytical superdex-200 column equilibrated with 20 mM Tris pH 8, 100 mM NaCl, 0.1% (w∶v) LAPAO, at a flow rate of 0.4 mL/min.

When necessary, pure proteins were concentrated on an Amicon concentrator with a 50 kDa cut-off.

For detergent to amphipol exchange, 5 g of amphipols were added per g of pure NTT1. The mixture was incubated for 1 to 2 hours at 4°C. BioBeads (Biorad) were then added four times to remove detergents, with each addition corresponding to a 10-fold weight-excess of the detergent initially present in solution.

### Activity measurements

Radioactive ATP transport was measured on whole *E. coli* cells as described previously [6]. ADP/ATP exchange was followed by luminescence on whole *E. coli* cells. After protein overexpression, cells were washed and resuspended in 50 mM Hepes buffer pH 7.5 at 50 µg/µL. Experiments were performed in 96-well plates. Cells (10 µL) were added to 90 µL of Hepes buffer containing 40 µM luciferin (Sigma) and 40 µg/mL luciferase (Sigma). Luminescence signal was continuously monitored in a luminometer with injectors. After 15 s of baseline recording, i) 10 µL of ADP stock solution are injected and the signal is still recorded for 60 s or, ii) 10 µL of Pi or buffer are injected 20 s after ADP and the signal is still acquired for 60 s. A reference curve corresponding to the addition of the same ADP concentration in buffer is subtracted to the curve obtained with the cells overexpressing the transporter. Comparison of transport rates in different conditions corresponds to the increase of luminescence signal during 20 s after ADP or Pi addition.

For all activity experiments, NTT1 forms were quantified using whole cell proteins separated by SDS-PAGE and transferred onto nitrocellulose membrane. NTT1 constructs were then detected with anti-His-tag peroxidase or *Strep*-Tactin peroxidase conjugates, depending on the construct used. Western-blots were revealed on a Kodak 4000 MM image station and densitometry quantification was performed with Molecular Imaging Software (Kodak). Different concentrations of pure NTT1 were used as standards. V_M_ and K_M_ were determined by fitting the experimental data to the Michaelis-Menten equation.

### Analytical ultracentrifugation

A Beckman XL-I analytical ultracentrifuge and an An-60Ti rotor (Beckman Coulter) with 12 mm or 3 mm optical path length cell equipped with sapphire windows were used for analytical ultracentrifugation. Absorbance at 280 nm and interference profiles were measured for 16 hours at 42,000 rpm and 10°C. Analysis was done in terms of continuous size-distribution (c(s)) with the Sedfit program [11], considering 200 particles with sedimentation coefficients, s, between 0.1 and 20 S, with a frictional ratio of 1.25 and a partial specific volume intermediate between that of NTT1 and that of the detergent used. The parameters used were: 132,000 L.mol^−1^.cm^−1^ for the ε of NTT1; 0.74, 1.002, 0.94, 0.87 (φ′) and 0.82 for the partial specific volumes of NTT1 (Sednterp), LAPAO [12], foscholine-12 (FC12) (Anatrace data), amphipols [13] and β-dodecylmaltoside (β-DDM) [14], respectively; and 0.187, 0.134, 0.14, 0.15 and 0.143 for the refractive index increment of NTT1, LAPAO [12], FC12 (Anatrace data), amphipols [13] and β-DDM [14], respectively. A regularization procedure was also applied with a confidence level of 0.68. Sample density and viscosity were 1.004 g/mL and 1.32 mPa.s, respectively, as determined with Sednterp.

### Electron microscopy

NTT1 SEC peak fractions were used for electron microscopy. Four microliters of protein sample, at approximately 0.05–0.1 mg/mL, were adsorbed onto the clean face of a carbon film on a mica sheet (carbon/mica interface) and negatively stained with 2% (w/v) neutral sodium silicotungstate. Micrographs were taken under low-dose conditions with a CM12 LaB6 electron microscope working at 120 kV and with a nominal magnification of 45,000×.

### Tryptophan fluorescence measurements

Tryptophan fluorescence emission spectra were measured at 25°C on a PTI quanta master 4 (Photon Technology International, London, ON, Canada). Emission spectra were recorded from 310 to 370 nm using an excitation wavelength of 295 nm, with a 2 nm excitation and a 4 nm emission band pass. The cuvette contained 1 mL of 0.2 µM NTT1, and increasing concentrations of ADP, ATP or ADP+ATP were added. Values were corrected for dilution and for the inner-filter effect of nucleotides using another cuvette containing 2.2 µM N-acetyltryptophanamide (NATA), as previously described [15]. Experimental data were fitted to the following equation:
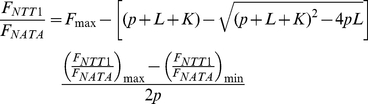
where *F* corresponds to the intensities of the various spectra analyzed, *p* is the concentration of NTT1 in the cuvette, *L* is the concentration of nucleotides added and *K* is the dissociation constant.

## Results and Discussion

### Purification and oligomeric status of NTT1 in surfactant solutions

**Figure 1:**
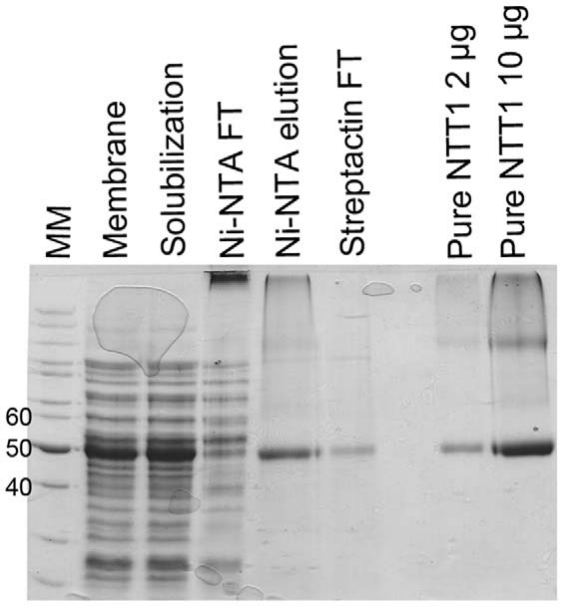
Purification of NTT1. SDS-PAGE analysis of the NTT1 purification procedure. MM, Molecular weight markers. FT, flow-through.

During import into the chloroplast, NTT1 becomes folded and functional only after cleavage of its N-terminal transit peptide. NTT1 was thus expressed in *E. coli* as a matured form (*i.e.* lacking this N-terminal part). The overexpressed protein inserted into the bacterial plasma membrane in a functional state, as previously shown using radioactive ATP import into bacteria [6] and ATP export measured by luminescence [6]. To further purify NTT1, a two-step affinity chromatography protocol was set up. The protein was first solubilized in the presence of LAPAO (Figure 1). Solubilized proteins were then loaded onto a Ni-NTA matrix, which is compatible with high detergent concentrations. Proteins eluted from the Ni-NTA matrix were further purified on a *Strep*-Tactin chromatography column (Figure 1). Using this protocol, highly pure NTT1 was obtained in sub-milligram amounts per liter of *E. coli* culture. The yield and purity of the purified NTT1 protein was markedly better than in previous studies using a different purification procedure [3]. The good purification yield is mainly due to the high expression levels reached using our combination of bacterial strain and constructs. In addition, LAPAO appears to solubilize NTT1 more efficiently from the bacterial membrane than β-DDM [6], which was the detergent used in other studies [3], [6].

**Figure 2:**
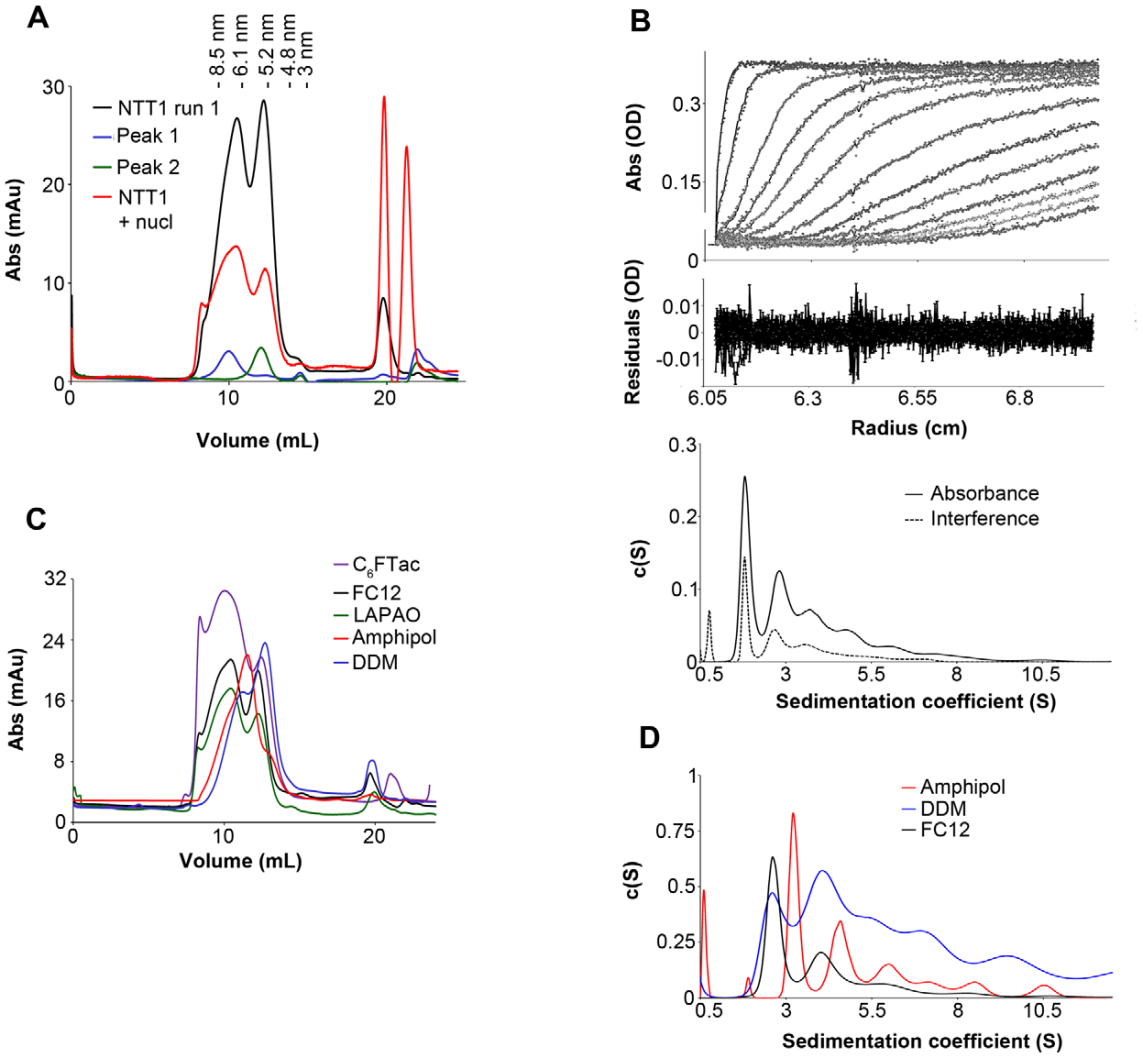
Analysis of the oligomeric status of NTT1 in various detergents. A, SEC elution profiles of NTT1 in LAPAO. Pure NTT1 first run (black line), second SEC run of elution peak 1 from the first run (10 mL, blue line), second SEC run of elution peak 2 from the first run (12 mL, green line) and pure NTT1 mixed with ADP, ATP and Pi (red line). Stokes radii of the standard proteins used to calibrate the column are indicated on top of the graph. B, analysis of sedimentation velocity of pure NTT1 in LAPAO. The upper part shows several time-points during sedimentation velocity experiments with the experimental points as markers and the fits of the data as lines. The central part highlights the residuals between experimental points and fits. The lower part presents the absorbance and interference c(S) distributions of NTT1 in LAPAO. C, SEC elution profile of pure NTT1 in different detergents: NTT1 in C_6_FTac (purple line), NTT1 in FC12 (black line), NTT1 in LAPAO (green line), NTT1 in amphipols (red line) and NTT1 in β-DDM (blue line). D, sedimentation velocity of NTT1 in different detergents. Absorbance c(S) distributions of NTT1 in amphipols (red line), NTT1 in β-DDM (blue line) and NTT1 in FC12 (black line).

We previously reported that NTT1 is present as a mixture of oligomers in LAPAO solution [6]. Herein, the oligomeric status of purified NTT1 was further assessed by size exclusion chromatography (SEC) and analytical ultracentrifugation (AUC) in order to identify the type of oligomers. Purified protein solubilized in LAPAO was recovered in two main peaks at around 10 mL and 12 mL on an analytical superdex-200 column (Figure 2A). These two peaks correspond to species with Stokes radii of 6.9 and 5.2 nm, respectively. Other species were also present in the solution at higher molecular weights, as observed by the broadening of the dimer peak and by the presence of a small peak around the void volume of the column (Figure 2A and C). These peaks were variable between protein batches in terms of intensity and broadening.

NTT1 was further characterized by analytical ultracentrifugation, which allows the oligomeric status of a protein to be determined, as well as the amount of detergent bound to any species present in a complex sample [16]. Analysis of the data revealed that NTT1 in LAPAO was mainly present in two oligomeric forms sedimenting at 1.9 S and 2.9 S (Figure 2B and Table 1). Interference data enabled the quantification of bound LAPAO at 1.9 g/g of NTT1, for both forms (Table 1). The combination of these ratios, the S values (Table 1) and the Stokes radii indicated that these two forms were compact globular monomers and dimers of NTT1, respectively. Higher oligomers present in solution could be trimers and/or tetramers, but analysis of data for these species was less accurate due to broader peaks. Complementary analyses using Multiple Angle Laser Light Scattering indicated that the main higher oligomer is a tetramer (not shown). Taken together, data from SEC and AUC analysis of NTT1 solubilized in LAPAO were consistent, indicating that the protein is mainly present as monomers and dimers in solution with some higher oligomers.

**Table 1:** AUC data analysis.

Surfactant (Ū)	Monomer (% of the three species) (S_exp-10°C_)	Dimer (% of the three species) (S_exp-10°C_)	Tetramer (% of the three species)	Ratio detergent/protein (g/g)
LAPAO (1.002)	42±4 **(1.9)**	35±2 **(2.9)**	23±4	1.9±0.4
FC12 (0.975)	47±5 **(2.7)**	35±2 **(4.1)**	18±4	1.6±0.1
Amphipol (0.87)	44±1 **(3.5)**	33±0 **(5.0)**	23±1	1.6±0.2
DDM (0.82)	46±14 **(3.7)**	29±12 **(5.6)**	n.d.	n.d.

Proportion of the three main species of NTT1 in different surfactants and average weight ratio of detergent to protein in the different surfactants. The experimental sedimentation coefficient at 10°C for monomers and dimers is indicated between brackets (n.d.: not determined due to poor data quality).

The co-existence of monomers and dimers might result from an equilibrium between the two species. Therefore we assayed monomer-dimer interconversion by reinjecting each species (SEC peak fraction) onto the same analytical superdex-200. Both samples eluted at the same elution volume (Figure 2A), indicating that these two forms are not in equilibrium but form two stable NTT1 oligomers. The effect of nucleotides on the oligomeric status of NTT1 was also assayed by SEC. Adding a mixture of ADP, ATP and Pi did not change the monomer-dimer ratio for NTT1 in LAPAO (Figure 2A).

The oligomeric status of NTT1 in other detergents was also assayed by SEC (Figure 2C). Detergents were either directly exchanged on-column or in-solution for amphipols, as described in the material and methods section. In foscholine-12 (FC12), NTT1 displayed similar behavior to that described in LAPAO. The monomer-dimer ratio was similar in the two detergents, but a lower proportion of large oligomers was detected in FC12 (Figure 2C). In β-dodecylmaltoside (β-DDM), NTT1 tended to dissociate into monomers (Figure 2C). In amphipols, peak resolution was poor, leading to one main peak eluting at a volume between the typical NTT1 monomer and dimer peaks (Figure 2C). Finally, the fluorinated surfactant C_6_FTac decreased the amount of NTT1 monomers and increased the number of dimers and higher oligomers, as shown by the broadening of the dimer peak (Figure 2C).

It is well known that detergent exchange on a SEC never leads to complete removal of the initial detergent. Therefore, to be sure of the relevance of differences in NTT1 oligomeric behavior, the whole purification procedure, including the step solubilizing protein from the bacterial membranes, was also performed in FC12 and β-DDM. The oligomeric status of NTT1 in these preparations was analyzed by AUC. As observed for SEC-detergent exchange analysis, NTT1 originally solubilized in FC12 was mainly recovered as monomers and dimers (Figure 2D and Table 1). AUC analysis showed that NTT1 in amphipols was also mainly present in monomeric and dimeric forms (Figure 2D and Table 1), but well-defined larger oligomers were also detected. When NTT1 was directly solubilized in β-DDM, the AUC analysis revealed more features than the SEC detergent exchange analysis. Indeed, the protein behaved poorly in β-DDM, forming a large variety of oligomeric forms from monomers to aggregates (Figure 2D and Table 1). Moreover, an additional species sedimenting at 2.7 S was hypothetically attributed to an unfolded monomer form. Interference analysis revealed that, in FC12 and amphipols, approximately 1.6 g of amphiphiles were bound per g of NTT1. Thus, the shift in the distribution of sedimentation coefficient between the different surfactants can be attributed to the difference in their partial specific volume.

Altogether, the results of these biochemical and biophysical characterizations demonstrate that the purified transporter is quite stable in both LAPAO and FC12 (with well defined oligomeric forms and few aggregates). However, protein binding to *Strep*-Tactin beads is weaker in FC12 solution. Thus, LAPAO is the most appropriate detergent for study of NTT1 in solution.

### Low resolution structural analysis by electron microscopy

**Figure 3:**
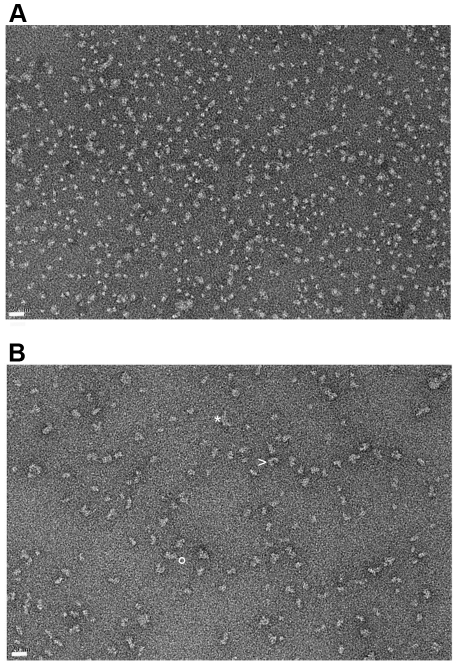
Electron microscopy analysis of NTT1. A, electron micrograph of NTT1 SEC monomer fraction (peak 2). The scale bar corresponds to 20 nm. B, electron micrograph of NTT1 SEC dimer fraction (peak 1). * indicates a typical fully extended dimer. > indicates U/V shaped particles. ° indicates densely packed dimers. The scale bar corresponds to 20 nm.

The SEC peak fractions of NTT1 monomers and dimers in LAPAO were harvested and subjected to negative staining electron microscopy. Both species displayed a well-defined shape (Figure 3A–B). Monomers formed very homogenous round particles of 5 to 10 nm diameter (Figure 3A), consistent with results from SEC and AUC experiments. Dimers were less homogenous and appeared as various particle shapes, including fully extended dimers, U/V shaped particles and densely packed dimers (Figure 3B). This could be the result of two monomers interacting through the base. The disparity of shape may be due to the extensive conformational changes that are possible in solution, and probably does not reflect behavior in lipid bilayers where lateral pressure would constrain the protein. Thus, electron microscopy indicates that both monomers and dimers formed stable and quite homogenous particles, with dimers exhibiting different conformations.

### Nucleotide binding to purified NTT1

When a tryptophan residue is located near to the ligand binding site of an enzyme or transporter, ligand binding can induce conformational changes resulting in measurable tryptophan fluorescence variations. The NTT1 construct used in this study contains 11 tryptophans; therefore, we explored this type of measurement to follow ADP and ATP binding to NTT1 in LAPAO solution.

**Figure 4:**
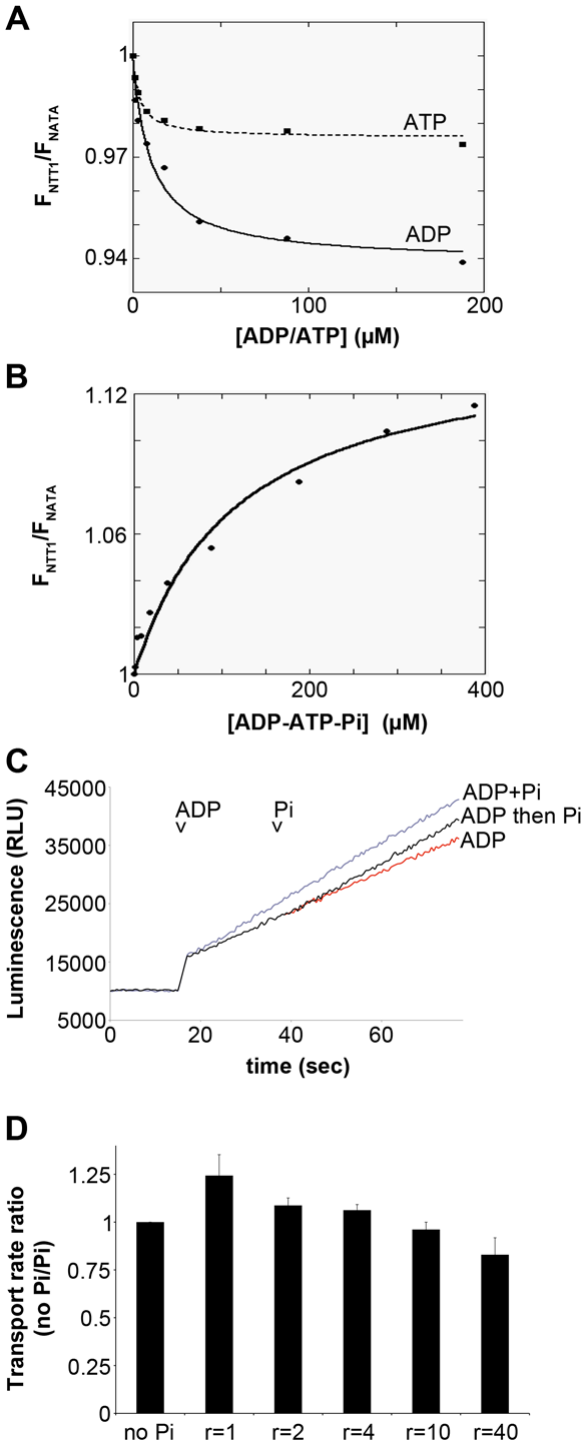
Nucleotide binding and Pi effect on NTT1. A, NTT1 tryptophan fluorescence quenching by ADP and ATP. For both experiments, points are represented with markers and fits with lines. Full line: ADP titration, dashed line: ATP titration. A typical experiment is presented. B, NTT1 tryptophan fluorescence upon titration with an equimolar ADP/ATP mixture in the presence of 1 mM Pi. Markers are experimental points and the continuous line corresponds to the fit. A typical experiment is presented. C, NTT1 transport activity followed by luminescence on whole *E. coli* cells expressing NTT1. “ADP”: ATP/ADP exchange in Hepes buffer, where 5 µM ADP was added at time t1 (ADP arrow). “ADP then Pi”: ATP/ADP exchange in Hepes buffer with sequential addition of 5 µM ADP at time t1 (ADP arrow) and 5 µM Pi at time t2 (Pi arrow). “ADP+Pi”: ATP/ADP exchange in Hepes buffer with simultaneous addition of 5 µM ADP and 5 µM Pi at time t1 (ADP arrow). D, Comparison of the transport rate at different Pi/ADP ratio (r) at 5 µM ADP. Transport rate without Pi is the reference and set to 1. For the panel A and B, the experiments have been performed three to five times with independent protein batches.

Titration of the purified NTT1 in LAPAO by either ADP or ATP resulted in tryptophan fluorescence quenching, with saturation at around 100 to 200 µM of ADP or ATP (Figure 4A). Fitting the experimental values with a single binding site equation led to the determination of 4 and 9 µM affinities for ATP and ADP, respectively (Table 2). These data are in good agreement with the previously published K_M_ values for NTT1 present in bacterial membranes [6], [17], suggesting that purified NTT1 in LAPAO is stable in these conditions, and that the binding site and probably also the protein as a whole are correctly folded. NTT1 tryptophan fluorescence was also titrated with an equimolar mixture of ADP and ATP. Data from this assay could be fitted with a monophasic equation, but not with a biphasic equation. The affinity was determined to be approximately 2 µM for the 1 to 1 ADP:ATP mixture (Table 2). These data therefore suggest that co-binding of ADP and ATP induces a slight cooperative effect indicative of two concerted binding-sites.

**Table 2:** Affinities of wild-type NTT1 for different nucleotide mixtures and effect of Mg^2+^ based on tryptophan fluorescence variations.

Nucleotide(s)	Affinity (µM)	Number of independent binding sites	Second site affinity (µM)	Mg^2+^ effect
ADP	8.6±1.5	1		Before titration: no ADP binding After titration: partial signal dequenching
ATP	4.2±0.7	1		Before titration: no ADP binding After titration: partial signal dequenching
ADP+ATP (equimolar)	2.3±0.15 (1.15 µM of each nucleotide)	1		
ADP+Pi (equimolar)	3.4±0.8	2	22.5±17.9	
ATP+Pi (equimolar)	8.7±1.0	1		
ADP+ATP (equimolar in the presence of 1 mM Pi)	117.2±30.0	1		

. Mg^2+^ effect was assessed by adding 1 mM MgCl_2_ before or after the complete titration of NTT1 with ADP or ATP. All the affinities were determined using the procedure described for the figure 4A.

To gain more insight into the nucleotide binding sites and the translocation mechanism, fluorescence measurements were performed in different conditions. The influence of Mg^2+^ on ATP or ADP binding was analyzed in two ways. Adding 1 mM Mg^2+^ before the nucleotides prevented fluorescence quenching, suggesting that it inhibited nucleotide binding to the protein (Table 2). In contrast, when Mg^2+^ was added after complete nucleotide titration, only half of the initial fluorescence was restored (Table 2), to a level equivalent to the addition of 5–10 µM ATP/ADP. This clearly indicates that nucleotides strongly bind to NTT1 and confirms that Mg^2+^-complexed nucleotides are not recognized by the transporter.

The influence of Pi in various conditions was also assayed as Pi has been described as a co-transported substrate of ADP [3]. Binding of equimolar amounts of ADP:Pi or ATP:Pi gave rise to calculated affinities of 3 and 9 µM, respectively (Table 2). Thus, Pi improved the affinity for ADP but decreased that for ATP. These results can be interpreted if we consider that there are two binding pockets on NTT1, one for ADP and Pi, the other for ATP. The presence of Pi could lock the ADP binding site in a more favorable position, as shown by the increased affinity, thus confirming the hypothesis that Pi is a co-substrate. The second site could strictly accommodate ATP, thus Pi binding would compete with one or several of the three phosphates, thereby decreasing affinity for ATP.

To further understand the effect of Pi, NTT1 was titrated with a 1 to 1 mixture of ADP:ATP in the presence of 1 mM Pi. In these conditions, the protein behavior was drastically changed. The titration still took place but the tryptophan fluorescence, rather than being quenched, increased to saturation (Figure 4B). Moreover, the amplitude of the fluorescence variation was far more important than in the case of the quenching in the absence of Pi. Fitting the data from this titration indicated to an affinity for ADP:ATP close to 120 µM (Table 2). A possible explanation for this change in fluorescence variation could be that, when Pi is added at the same time as ADP+ATP, it enables nucleotide transport. This means that nucleotides would not remain bound to NTT1, and NTT1 would undergo conformational changes during which the relative positions and orientations of tryptophans towards nucleotides would vary significantly as indicated by the very different behavior of their intrinsic fluorescence.

### Influence of Pi on nucleotide transport

Until further knowledge on the structure of the binding sites and the relative tryptophan locations has been acquired any explanation of these tryptophan fluorescence variations will remain speculative. Nevertheless, the very peculiar effect of Pi and its role in nucleotide transport were further studied through additional experiments. We measured transport rates using a luminescence assay recently developed in our laboratory [6]. The main advantage of this assay over measurement of radioactive ATP uptake, which is the current method [2], [6], [7], [17], [18], is that it makes it possible to follow the transport kinetics in real-time, with very good temporal resolution. It also allows sequential addition of compounds and direct observation of their effects.

Generally, ATP/ADP exchange assays were performed in phosphate buffer, which provides extensive amounts of Pi (50 mM). In these conditions, the luminescence signal increased constantly at a significant rate, indicating that transport was fast and efficient [6]. To deprive ADP of its co-substrate, bacterial cultures were resuspended in Hepes buffer instead of phosphate buffer. In this condition, the luminescence signal continuously increased over time, demonstrating that ATP/ADP exchange still occurs without Pi (Figure 4C, ADP curve). Simultaneous addition of ADP and Pi at 5 µM in Hepes buffer led to slightly faster transport (24+/−10% increase, average of 10 measurements) than after addition of ADP alone (Figure 4C, ADP+Pi curve). Similarly, when ATP/ADP exchange was already initiated in Hepes buffer, the subsequent addition of 5 µM Pi also increased the transport rate (Figure 4C, ADP then Pi curve). In order, to further characterize the effect of Pi, the transport rates were compared at different Pi/ADP ratio. The higher rate is obtained at a 1 to 1 Pi/ADP ratio, while at ratio higher than 10, the transport is even slower than in ADP only condition.

In conclusion, Pi was confirmed to be co-transported with ADP in equimolar amounts, but a high Pi concentration is also able to inhibit the transport suggesting a regulating role for this molecule in the chloroplast. Thus, NTT1 probably takes up one ATP molecule in exchange of one molecule of ADP and one molecule of Pi per translocation cycle. Therefore, the influence of Pi on fluorescence and transport experiments also suggests that the transporter's translocation cycle is triggered by the simultaneous presence of Pi, ADP and ATP.

### ADP/ATP binding to NTT1 mutants with altered activity

Several NTT1 mutants have been described with impaired transport activity [8]. Of these, we produced K155E, K155R, E245K, K527E and K527R in *E. coli*, and assessed their activities by radioactive ATP uptake (Figure S1). The different mutants were then purified and ADP/ATP binding was monitored by tryptophan fluorescence variation measurements (Table 3). K155E was no longer able to bind either ADP or ATP (Table 3). K155R was not able to bind ADP and had a very low affinity (around 100 µM) for ATP (Table 3). These results were in good agreement with the low residual transport rate of all the K155 mutants (Figure S1) and indicate that lysine 155 is probably involved in ADP binding. K155E was also the only mutant which was mainly recovered as a dimer in solution (Table 3, AUC). Thus, this mutation might also induce more extensive structural changes.

**Table 3:** Characteristics of NTT1 mutants purified in LAPAO. Affinities for ADP and ATP, protein stability and oligomeric status.

Mutant	Affinity for ADP (µM)	Affinity for ATP (µM)	Protein properties	Oligomeric status
K155E	n.b.	n.b.		More dimer
K155R	n.b.	91.2±58.8		Similar to WT
E245K	14.0±5.1	1.9±1.7		Similar to WT
K527E*	19.0±24.1	n.b.		Similar to WT
K527R*	33.5±22.3	>1 mM	Less stable	Similar to WT
NTT1ΔC	Site 1: 0.6±0.7 Site 2: 31.1±28.2	0.7±0.5 25.1±13.1	Limited protein stability	Many large oligomers and/or aggregates

n.b.: no binding. The affinities were determined by tryptophan fluorescence variation measurements as described for the figure 4A. The oligomeric status was assessed by AUC experiments using the purified mutants in LAPAO.

* these mutants are equivalent to the K446 mutants used in [3].

In contrast, E245K was almost incapable of ATP/ADP exchange across the bacterial membrane (Figure S1) but maintained an affinity for ADP of around 14 µM (Table 3). NTT1-E245K was also clearly able to bind ATP, but the fluorescence variations were difficult to fit reliably. The curve appeared to indicate a biphasic binding mode with an affinity of 2 µM for the high affinity “site”. Altogether, these data suggest that glutamate 245 is involved in the translocation mechanism rather than in nucleotide binding.

ADP binding was similar for both K527E and K527R mutants, with affinities of about 20–35 µM, which is about three times lower than the affinity of the wild-type protein. In contrast, K527E was unable to bind ATP and K527R only had a very low ATP affinity (above 1 mM) [Table 3]. Thus, lysine 527 is probably involved in ATP binding. K527E exhibited only a low transport activity (Figure S1), while K527R retained some ATP import activity (Figure S1). This contrasts with results obtained in previous studies [8]. ATP import by NTT1-K527R is probably due to the presence of mM concentrations of ADP in *E. coli* cells that force or enhance transport, while K527E, which has no affinity for ATP, is blocked in an ADP bound form. These results confirm the conclusion that NTT1 functions in a counter exchange transport mode, in good agreement with the ADP/ATP/Pi binding properties measured on the wild-type protein. The importance of Pi was also indicated by transport measurements.

Altogether, nucleotide binding studies allowed us to decipher two different surfaces of the transporter with two distinct, but probably not independent, binding sites. Thus, K155 would be located in the ADP binding site, while K527 would be part of the ATP binding site. However, allosteric effects influence both binding sites since mutations of both these sites abolished not only the interaction with the respective nucleotide, but also affected the affinity of NTT1 for the other nucleotide. Finally, the conformational changes required to switch between the different transporting states of NTT1 involve charged residues such as E245.

### C-terminal fusion and deletion: impact on activity, oligomeric state and nucleotide binding

We recently observed that an N-terminal Mistic fusion to NTT1 (misNTT1) altered its transport activity [6] (Table 4). Thus, to gain further insight into NTT1 transport properties, a C-terminal Mistic fusion to NTT1 (termed NTT1mis) was also designed during the present work. The activity of this fusion protein was strikingly different to that of the native protein and of misNTT1, with a specific activity, at 50 µM ATP, which is almost 100-fold that of NTT1 (Table 4). To further characterize the transport rate of this NTT1mis C-terminal fusion protein, V_M_ and K_M_ values were determined using radioactive ATP uptake and luciferase assays (Table 4). The K_M_ values measured for ADP and ATP were 12 µM and 13 µM, respectively (Table 4 and Figure S2), thus in the same range for ADP, and around three times lower for ATP compared to the values obtained for NTT1. In contrast, the V_M_ value was 40 times higher for the C-terminal fusion than for NTT1 (Table 4). Thus, fusion proteins with Mistic placed at either the N- or C-terminal of NTT1 have opposing effects on NTT1 transport activity. A possible hypothesis explaining the effect of Mistic located at the C-terminus, could be that fusion induced oligomerization, as was observed for the N-terminal Mistic fusion protein [6]. To determine whether NTT1 activity was enhanced by C-terminal-triggered oligomerization, a C-terminal GFP fusion protein was also designed. Indeed, GFP is a soluble protein known to dimerize [19]. The activity of this C-terminal GFP fusion was only two-fold that of the unfused protein (Table 4), which is significantly less impressive than the C-terminal Mistic fusion protein. Thus, inducing close contact of transporter molecules due to modifications near the C-terminus increases the transport rate, but is perhaps not the main cause of this effect.

**Table 4:** Comparison of transport activities for different NTT1 fusions and deletion.

Construct	Relative NTT1 activity (%)	V_M-ATP_ (nmol ATP/min/mg NTT1)	K_M-ATP_ (µM)	K_M-ADP_ (µM)
NTT1 from [6]	100	10.1±2.5	32±6.0	6.7±2.4
NTT1mis	9300	408.2±74	13.2±5.3	12.0±5.2
NTT1GFP	200.2	-	-	-
NTT1ΔC	1236.2	-	-	-
misNTT1 from [6]	16.4	1.2±0.7	40.2±16.7	19.2±8.6

Relative NTT1-WT activities were determined from specific radioactive ATP uptake (using 50 µM ATP) performed during 5 minutes for wild type (WT) NTT1 and for mutants NTT1-K155E, NTT1-K155R, NTT1-E245K, NTT1-K527E and NTT1-K527R. The activity of WT is defined as 100% activity. The V_M_ and K_M_ values for ATP and the K_M_ value for ADP were determined as described in [6] using the radioactive ATP uptake method for ATP and the luminescence approach for ADP. Each measurement is the mean of three independent experiments.

**Figure 5:**
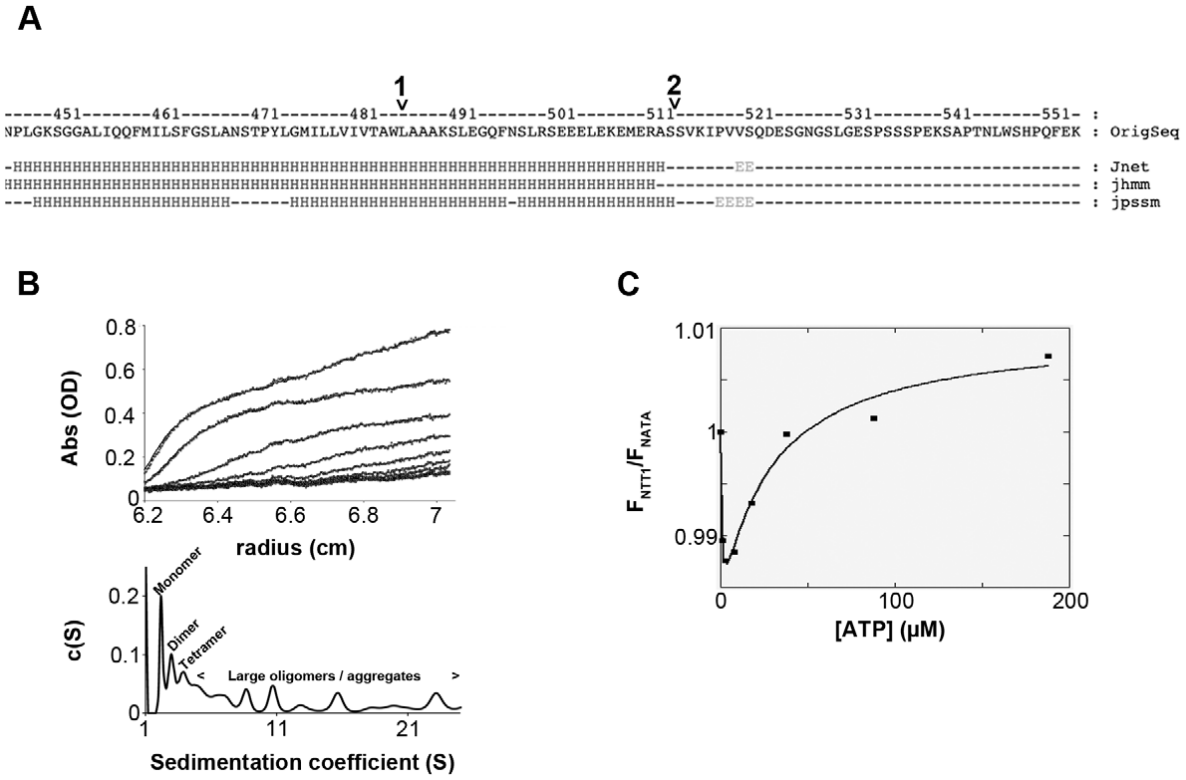
Properties of C-terminal deleted NTT1 (NTT1ΔC). A, sequence and secondary structure prediction of NTT1 C-terminus. The secondary structure prediction was obtained using the Jpred server [21]. Jnet is the predicted secondary structure. Jhmm is the predicted Jnet hmm profile. Jpssm is the predicted Jnet PSIBLAST pssm profile. The first arrow indicates the predicted end of the last NTT1 transmembrane helix (TMHMM pred). The second arrow indicates the end of the NTT1 C-terminal deletion construct, which also corresponds to the cleavage point during limited proteolysis treatment. B, sedimentation velocity analysis for NTT1ΔC. The upper part shows experimental points (markers) and fits (lines) of the sedimentation velocity experiments. The lower part corresponds to the absorbance c(S) distribution for NTT1ΔC. C, typical tryptophan fluorescence titration by ATP for NTT1ΔC. Markers show the experimental points and the continuous line is the fit with a two binding-sites equation.

N-terminal sequencing of purified NTT1 solution after limited proteolysis previously showed that the N-terminus can be cleaved-off [6]. Furthermore, N-terminal sequencing of the proteolyzed solution led to the identification of a C-terminal product corresponding to the 41 last residues of NTT1. Another interesting feature of this C-terminal domain is that it is predicted to be unstructured (Figure 5A, part after the second arrow) and is located at the end of the helical domain following the last predicted transmembrane helix (Figure 5A first arrow and [8]). Because of these observations, we deleted the last 41 NTT1 amino-acids to generate the corresponding construct (named NTT1ΔC). This protein was used to assess the effect of a C-terminal deletion on transporter activity. The specific activity of this construct was more than 10-fold that of the full-length NTT1 (Table 4). The construct was well expressed in *E. coli* and could be purified for study of its oligomerization behavior and nucleotide binding properties. However, the purified transporter had an unexpected tendency to aggregate (Figure 5B). Despite this, the correctly folded fraction of the protein was found to be composed predominantly of monomer and dimer species. ADP and ATP binding properties were investigated by tryptophan fluorescence variation. Both nucleotides bound NTT1ΔC with a biphasic curve (the ATP titration curve is presented in Figure 5C). After initial fluorescence quenching in the low micromolar range for substrates, fluorescence then increased up to nucleotide concentrations in the hundreds of micromolar range. The binding curves were fitted with a two-independent binding site equation and gave affinities for both ADP and ATP of about 0.6–0.7 µM and 25–30 µM for the first and second sites, respectively (Table 3). Thus, deleting the C-terminus of NTT1 has a significant impact on transport rate, but also affects the stability/oligomerization state of the protein, as well as its nucleotide binding abilities.

To conclude, C-terminal fusions or deletions of NTT1 increased its transport activity. Thus, it is possible that the flexible NTT1 C-terminus is an internal regulator domain of the nucleotide exchange rate. Furthermore, this domain also influenced the stability of the protein in solution probably *via* intramolecular interactions.

### Conclusions - Perspectives

In this work, we performed a detailed molecular characterization of the chloroplast envelope ATP/ADP transporter from *A. thaliana*. We used various biophysical methods to determine the oligomeric status of NTT1 and the affinities of the purified transporter for its substrates. This information provided us with some insights into the transport mechanism.

Through different approaches, we confirmed that NTT1 counter exchanges ADP+Pi against ATP as initially shown by Trentmann and co-workers [3]. Indeed, we have shown that Pi is strictly required to achieve steady-state transport rates.

However, in contrast to this study, our data conduct to an alternative model for NTT1 nucleotide transport. Indeed, nucleotide binding to NTT1 highlighted the presence of two distinct binding sites for ADP+Pi and for ATP, respectively, which were shown to be allosterically coupled. Based on these results, the functional unit of NTT1 could either be a monomer with concerted motion for nucleotide translocation, or a dimer (or higher oligomers such as a tetramer) that achieves translocation through a concerted-protomer motion producing a cooperative transport mechanism. This hypothesis is consistent with the results obtained on transport activity for NTT1 constructs N-terminally fused to Mistic [6]. Indeed, fusion of Mistic to NTT1's N-terminus induces the formation of large oligomers with low ATP/ADP exchange rates. Formation of these large oligomers is driven by Mistic boundaries and is probably incompatible with NTT1's coordinated transport [6]. In support of this, a high propensity to form small sized-oligomers was shown to favor high transport rates [6]. Furthermore, in the present study we assessed, for the first time, the oligomeric status of NTT1 in different amphiphiles. On one hand, fluorinated surfactants are known to favor physiological oligomeric status [20] and induce the conversion of NTT1 monomers to dimers and higher oligomers. On the other hand, detergents are well known to dissociate membrane protein complexes and oligomers and mainly lead to monomers and dimers. Thus, it is likely that the dimers of NTT1 are partly dissociated upon solubilization.

Finally, we showed that *in vitro* this transporter is negatively regulated by its flexible C-terminal end.

Altogether these data support the presence of an active, functional NTT1 dimer providing concerted ADP-Pi/ATP exchange. These first insights on the oligomeric status and the function of NTT1 are a first breakthrough towards complete functional and molecular characterization of this transporter.

## Supporting Information

Figure S1 **Transport activity of NTT1 mutants.** Specific radioactive ATP uptake (using 50 µM ATP) was measured at 5 minutes for wild type (WT) NTT1 and for mutants NTT1-K155E, NTT1-K155R, NTT1-E245K, NTT1-K527E and NTT1-K527R. The activity of WT is defined as 100% activity. Each measurement is the mean of three independent experiments.
(TIF)

Figure S2 **Determination of K_M_ of NTT1 for ADP by luminescence.** Typical Michaelis-Menten curve allowing the determination of K_M_ of NTT1 for ADP, based on a luminescence experiment. Experimental points are shown as dots and the continuous line corresponds to the fit. The experiments have been performed three times with independent cell batches.
(TIF)

Table S1 **Oligonucleotide sequences of the different primers used for cloning.**
(DOC)

## References

[1] Linka N, Weber AP (2010). Intracellular metabolite transporters in plants.. Mol Plant.

[2] Neuhaus HE, Thom E, Mohlmann T, Steup M, Kampfenkel K (1997). Characterization of a novel eukaryotic ATP/ADP translocator located in the plastid envelope of Arabidopsis thaliana L.. Plant J.

[3] Trentmann O, Jung B, Neuhaus HE, Haferkamp I (2008). Nonmitochondrial ATP/ADP transporters accept phosphate as third substrate.. J Biol Chem.

[4] Haferkamp I, Fernie AR, Neuhaus HE (2011). Adenine nucleotide transport in plants: much more than a mitochondrial issue.. Trends Plant Sci.

[5] Mohlmann T, Tjaden J, Schwoppe C, Winkler HH, Kampfenkel K (1998). Occurrence of two plastidic ATP/ADP transporters in Arabidopsis thaliana L.–molecular characterisation and comparative structural analysis of similar ATP/ADP translocators from plastids and Rickettsia prowazekii.. Eur J Biochem.

[6] Deniaud A, Bernaudat F, Frelet-Barrand A, Juillan-Binard C, Vernet T (2011). Expression of a chloroplast ATP/ADP transporter in E. coli membranes: Behind the Mistic strategy.. Biochim Biophys Acta.

[7] Frelet-Barrand A, Boutigny S, Moyet L, Deniaud A, Seigneurin-Berny D (2010). Lactococcus lactis, an alternative system for functional expression of peripheral and intrinsic Arabidopsis membrane proteins.. PLoS One.

[8] Trentmann O, Decker C, Winkler HH, Neuhaus HE (2000). Charged amino-acid residues in transmembrane domains of the plastidic ATP/ADP transporter from arabidopsis are important for transport efficiency, substrate specificity, and counter exchange properties.. Eur J Biochem.

[9] Trentmann O, Horn M, van Scheltinga AC, Neuhaus HE, Haferkamp I (2007). Enlightening energy parasitism by analysis of an ATP/ADP transporter from chlamydiae.. PLoS Biol.

[10] van den Ent F, Lowe J (2006). RF cloning: a restriction-free method for inserting target genes into plasmids.. J Biochem Biophys Methods.

[11] Schuck P, Rossmanith P (2000). Determination of the sedimentation coefficient distribution by least-squares boundary modeling.. Biopolymers.

[12] Nury H, Manon F, Arnou B, le Maire M, Pebay-Peyroula E (2008). Mitochondrial bovine ADP/ATP carrier in detergent is predominantly monomeric but also forms multimeric species.. Biochemistry.

[13] Gohon Y, Pavlov G, Timmins P, Tribet C, Popot JL (2004). Partial specific volume and solvent interactions of amphipol A8-35.. Anal Biochem.

[14] Salvay AG, Santamaria M, le Maire M, Ebel C (2007). Analytical ultracentrifugation sedimentation velocity for the characterization of detergent-solubilized membrane proteins Ca++-ATPase and ExbB.. J Biol Phys.

[15] Divita G, Di Pietro A, Roux B, Gautheron DC (1992). Differential nucleotide binding to catalytic and noncatalytic sites and related conformational changes involving alpha/beta-subunit interactions as monitored by sensitive intrinsic fluorescence in Schizosaccharomyces pombe mitochondrial F1.. Biochemistry.

[16] le Maire M, Arnou B, Olesen C, Georgin D, Ebel C (2008). Gel chromatography and analytical ultracentrifugation to determine the extent of detergent binding and aggregation, and Stokes radius of membrane proteins using sarcoplasmic reticulum Ca2+-ATPase as an example.. Nat Protoc.

[17] Tjaden J, Schwoppe C, Mohlmann T, Quick PW, Neuhaus HE (1998). Expression of a plastidic ATP/ADP transporter gene in Escherichia coli leads to a functional adenine nucleotide transport system in the bacterial cytoplasmic membrane.. J Biol Chem.

[18] Thuswaldner S, Lagerstedt JO, Rojas-Stutz M, Bouhidel K, Der C (2007). Identification, expression, and functional analyses of a thylakoid ATP/ADP carrier from Arabidopsis.. J Biol Chem.

[19] Yang F, Moss LG, Phillips GN (1996). The molecular structure of green fluorescent protein.. Nat Biotechnol.

[20] Breyton C, Pucci B, Popot JL (2010). Amphipols and fluorinated surfactants: Two alternatives to detergents for studying membrane proteins in vitro.. Methods Mol Biol.

[21] Cole C, Barber JD, Barton GJ (2008). The Jpred 3 secondary structure prediction server.. Nucleic Acids Res.

