# Supplementary figures and images for: Oligomeric Status and Nucleotide Binding Properties of the Plastid ATP/ADP Transporter 1: Toward a Molecular Understanding of the Transport Mechanism
Source: PLoS One. 2012 Mar 16;7(3):e32325. doi: 10.1371/journal.pone.0032325 (PMC3306366; doi:10.1371/journal.pone.0032325)

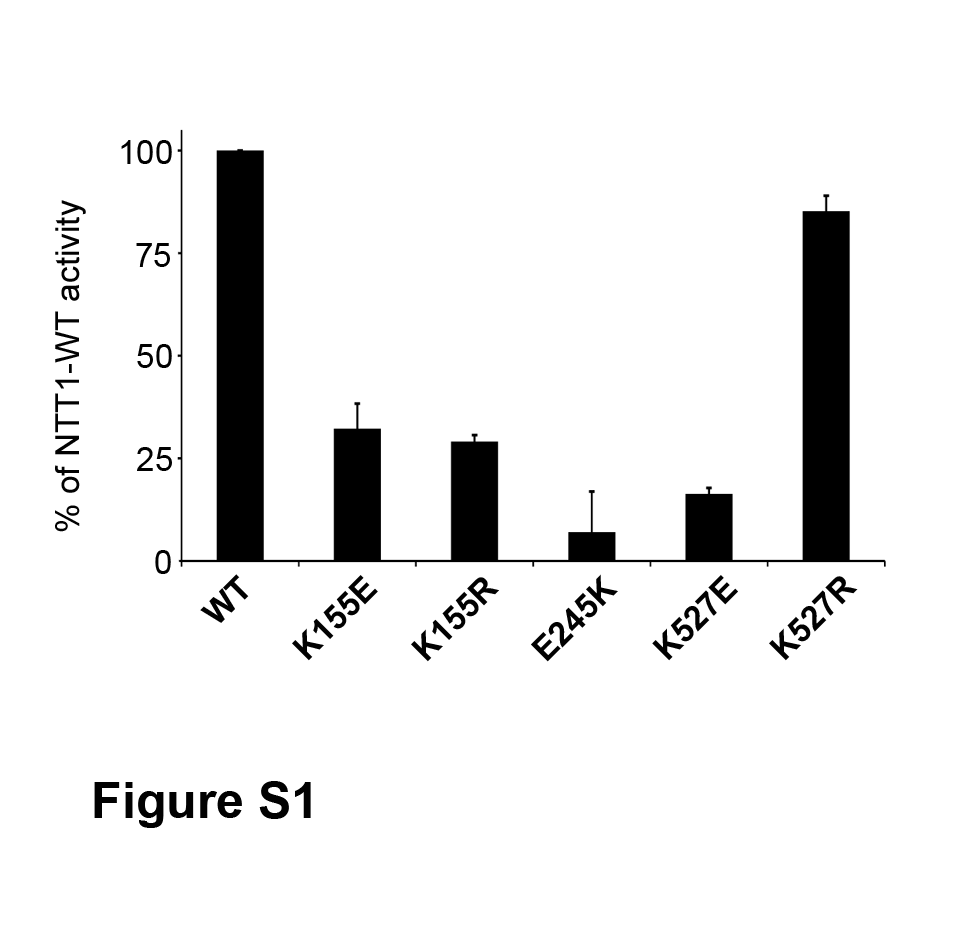

Supplement: Figure S1 — Transport activity of NTT1 mutants. Specific radioactive ATP uptake (using 50 µM ATP) was measured at 5 minutes for wild type (WT) NTT1 and for mutants NTT1-K155E, NTT1-K155R, NTT1-E245K, NTT1-K527E and NTT1-K527R. The activity of WT is defined as 100% activity. Each measurement is the mean of three independent experiments. (TIF) [file pone.0032325.s001.tif]
