# Supplementary material for: Oligomeric Status and Nucleotide Binding Properties of the Plastid ATP/ADP Transporter 1: Toward a Molecular Understanding of the Transport Mechanism
Source: PLoS One. 2012 Mar 16;7(3):e32325. doi: 10.1371/journal.pone.0032325 (PMC3306366; doi:10.1371/journal.pone.0032325)
Supplement: Table S1 — Oligonucleotide sequences of the different primers used for cloning. (DOC) [file pone.0032325.s003.doc]

| **Primer Name** | **Nucleotide sequence** |
| --- | --- |
| K155Efor | ATA CCT TTC TTG GAG ACT TGG GTG |
| K155Erev | CAC CCA AGT CTC CAA GAA AGG TAT |
| K155Rfor | ATA CCT TTC TTG CGG ACT TGG GTG |
| K155Rrev | CAC CCA AGT CCG CAA GAA AGG TAT |
| E245Kfor | TAT GTT ATG GCT AAG CTT TGG GGT AG |
| E245Krev | CTA CCC CAA AGC TTA GCC ATA ACA TA |
| K527Efor | AAC CCA TTA GGG GAA TCA GGG GGA GCT TT |
| K527Erev | AAA GCT CCC CCT GAT TCC CCT AAT GGG TT |
| K527Rfor | AAC CCA TTA GGG CGA TCA GGG GGA GCT T |
| K527Rrev | AAG CTC CCC CTG ATC GCC CTA ATG GGT T |
| Cterfor | atg gag aga gct tca TAG GAC CCA GCT TTC |
| Cterrev | GAA AGC TGG GTC CTA TGA AGC TCT CTC CAT |
| MisticCterfor | GCT TTC TTG TAC AAA GTG GTC ATG TTT TGT ACA TTT TTT GAA AAA CAT CAC C |
| MisticCterrev | GCT TTG TTA GCA GCC TCG AAT CA TCA TT CTT TTT CTC CTT CTT CAG ATA CTG |
| GFPfor | gct ttc ttg tac aaa gtg gtC atg agt aaa gga gaa gaa ctt ttc |
| GFPrev | GCT TTG TTA GCA GCC TCG AAT CAT CAT TAT TTG TAG AGC TCA TCC |

**Table S1. Oligonucleotide sequences of the different primers used for cloning.**
